# Donor Variability and PD-1 Expression Limit BK Polyomavirus-specific T-cell Function and Therapy

**DOI:** 10.1097/TP.0000000000005399

**Published:** 2025-04-09

**Authors:** Maud Wilhelm, Amandeep Kaur, Anne Geng, Marion Wernli, Hans H. Hirsch

**Affiliations:** 1 Transplantation and Clinical Virology, Department of Biomedicine, University of Basel, Basel, Switzerland.

## Abstract

**Background.:**

BK polyomavirus (BKPyV) nephropathy is a major cause of premature kidney transplant failure. Current management relies on reducing immunosuppression to restore BKPyV-specific immune control. Ex vivo expansion and transfer of BKPyV-specific cytotoxic T cells prepared from third-party donors may enhance virus-specific treatment, but the efficacy seems suboptimal.

**Methods.:**

To optimize BKPyV-specific T-cell expansion protocols, we compared conventional and G-Rex expansion cultures at 10 and 14 d after stimulation with BKPyV overlapping peptide pools. Cytokine and cytotoxic responses were assessed as well as programmed cell death protein 1 (PD-1) and programmed cell death ligand 1 (PD-1L) expression on effector and target cells, respectively.

**Results.:**

Despite all donors being BKPyV-IgG seropositive, BKPyV-specific T-cell responses were heterogeneous and varied in magnitude between individuals. Overall, we observed higher cell counts in G-Rex compared to conventional cultures. Upon restimulation with 15mer-pools or immunodominant 9mer-pools, expanded BKPyV-specific T cells expressed polyfunctional markers, for example, interferon-γ, tumor necrosis factor-α and CD107a, and were cytotoxic for 9mP-pulsed autologous phytohemagglutinin blasts or BKPyV-infected allogeneic renal proximal tubule epithelial cells (RPTECs). Compared with conventional cultures, G-Rex-expanded CD4 and CD8 T cells showed higher PD-1 expression. Pembrolizumab reduced PD-1 expression on BKPyV-specific T cells and augmented polyfunctional BKPyV-specific T-cell responses and cytotoxicity. Interferon-𝛾 increased PD-L1 expression on BKPyV-infected RPTECs and increased viability.

**Conclusions.:**

Upregulated PD-1 expression of ex vivo expanded T cells contributes to third-party donor variability and potentially impairs the efficacy of adoptive T-cell therapy. Because BKPyV-infected RPTECs increase PD-L1 under inflammatory conditions, adding immune checkpoint inhibitors ex vivo before infusion could be evaluated for enhanced clinical efficacy when attempting treatment of BKPyV-associated pathologies without jeopardizing transplantation outcomes.

## Graphical abstract

**Figure:**
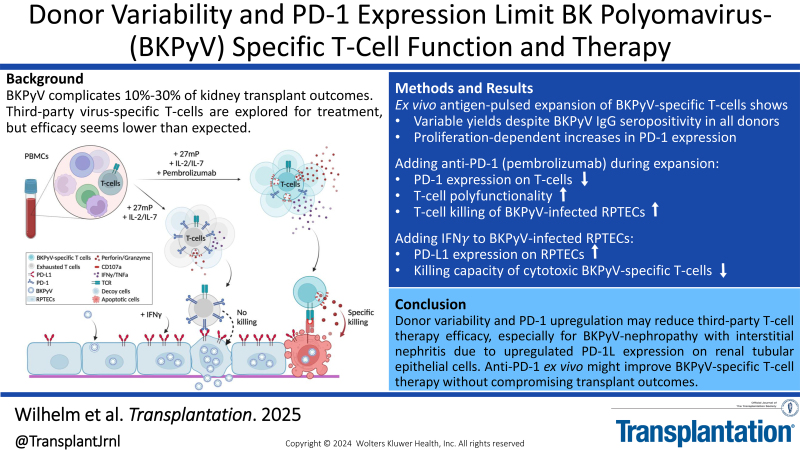

## Introduction

BK polyomavirus (BKPyV) infects >90% of the general population^1^ and thereafter persists in the renourinary tract without known ill effects, despite asymptomatic low-level viruria in approximately 10% of seropositive people.^2^ In immunodeficient persons, BKPyV can cause 3 major diseases, BKPyV-associated hemorrhagic cystitis in 5%–25% of allogeneic cell transplantation recipients^3,4^; BKPyV-associated nephropathy in 1%–15% of kidney transplant recipients (KTRs) and urothelial carcinoma in 0.1%–1% of mostly KTRs with prolonged uncontrolled BKPyV replication.^5^ Notably, BKPyV affects kidney transplant outcomes by directly and indirectly contributing to premature allograft failure.^6^ The shortened graft survival impacts the quality of life and puts significant stress on transplant programs, augments the burden of organ-donor shortage, and raises the risk of death while waitlisted for retransplantation.^6^ Estimates from large multicenter studies and registry analyses suggest that each year approximately 5000–10 000 newly transplanted KTRs experience significant BKPyV-DNAemia/nephropathy during the first year posttransplantation.^7-9^

Low-level viruria in immunocompetent persons emphasizes the ability of BKPyV to escape from virus-specific immune control already in the absence of immunosuppression.^10,11^ Furthermore, viruria in kidney transplant donors represents a significant risk factor for new-onset BKPyV-DNAemia and biopsy-proven BKPyV-nephropathy of KTRs.^12-14^ After kidney transplantation, 20%–40% of KTRs develop high-level viruria with urine BKPyV loads >10 million c/mL or decoy cell shedding; about half of these patients progress to BKPyV-DNAemia and nephropathy, initially without and then with failing renal transplant function.^9,15,16^ Because of the lack of effective antiviral drugs, current management recommends reconstituting BKPyV-specific immune control, facilitated by reducing maintenance immunosuppression.^6,17^ However, there is little evidence from randomized controlled studies comparing different protocols of reducing immunosuppression on clearance of BK polyomavirus-DNAemia and/or nephropathy as a primary outcome.^18,19^

Clearance of BKPyV-DNAemia/nephropathy has been shown to be associated with increasing BKPyV-specific T-cell responses,^20-22^ to which cytotoxic T cells contribute as effectors.^23,24^ Because frequencies of BKPyV-specific T cells are low in the peripheral blood of healthy donors and KTRs,^2,25^ we and others developed protocols for ex vivo expansion using overlapping peptides covering the BKPyV-encoded regulatory large T-antigen (LTag) and Vp1 capsid proteins.^20-22^ Using mature monocyte-derived dendritic cells and activated monocytes as antigen-presenting cells, we reported that BKPyV-specific polyfunctional and cytotoxic effector memory T cells could be expanded in conventional culture when using overlapping 27mer peptides of immunodominant 9mers present in the LTag.^26^ To optimize the yield of ex vivo expansion for functional characterization and adoptive T-cell therapy, we now compared the conventional culture with the commercially available G-Rex system, which has been used for rapid expansion of multivalent virus-specific T cells (multi-VST) from third-party donors.^27-30^ However, despite highly promising case series and limited evidence from phase II studies investigating tolerability and safety as primary endpoint, randomized controlled trials phase III studies are awaited to demonstrate the prophylactic, preemptive or therapeutic efficacy of third-party multi-VSTs. Here, we report that ex vivo expansion of BKPyV-specific T cells can provide higher yields but is highly donor-dependent and includes upregulated PD-1 expression, which may impair the clinical efficacy of adoptive T-cell therapy for KTRs. Importantly, we observed that BKPyV-infected primary human renal proximal tubule epithelial cells (RPTECs), the major pathology correlate of BKPyV-nephropathy, upregulate programmed cell death ligand 1 (PD-L1) in response to interferon-γ (IFN-γ).

## Materials and Methods

### Healthy Blood Donors

Peripheral blood mononuclear cells (PBMCs) were isolated from blood samples of 9 healthy donors (**Table S1, SDC**, http://links.lww.com/TP/D252) as approved by the ethics committee of both Basel Cantons (ECBB 267/06) and either used directly or after cryopreservation as described.^20^ The donor age ranged from 25 to 65 y (median 35 y) as is commonly found in blood donation centers and consisted of 25% males and 75% females. HLA typing was performed by next-generation sequencing using TruSight-HLA version 2 sequencing Panel (Illumina, San Diego, CA) and Miniseq high-output reagent kit (300 cycles) (Illumina, FC-420-1003). BKPyV and JC polyomavirus (JCPyV) serostatus were determined using virus-like particles-based IgG enzyme-linked immunosorbent assay as described previously.^23^

### BKPyV-derived Peptides, Conventional and G-Rex Cell Culture

Further information on peptide pools, PBMC isolation, cell media, reagents and antibodies are provided in the **Supplemental Methods** (**SDC**, http://links.lww.com/TP/D252). For conventional culture, 3 × 10^6^ PBMCs were seeded in 24-well plates in 1.5-mL R5 medium. On day 1, cells were stimulated with 27 mP at 200 ng/mL per peptide. On days 4 and 11, interleukin (IL)-2 (20 U/mL, PeproTech, 200-02) and IL-7 (5 ng/mL, PeproTech, 200-07) were added. On day 8, half of the medium was replaced, and cells were re-pulsed with 27 mP at 200 ng/mL. On day 14, cells were harvested, counted, and analyzed.

For G-Rex culture, 3 × 10^6^ PBMCs were seeded in the G-Rex vessel in 6 mL of the R5 medium and stimulated with 27 mP at 200 ng/mL. On days 4 and 11, IL-2 (20 U/mL) and IL-7 (5 ng/mL) were added to the cell culture. On day 7, half of the medium was replaced and cells were repulsed with 27 mP at 200 ng/mL. Cells were harvested on days 10 and 14 for analysis.

### Flow Cytometry Analysis

Cells were rechallenged with the indicated peptides (0.5 µg/mL, Eurogentec) for 6 h in the presence of Golgi stop (Becton Dickinson, Franklin Lakes, NJ, 554715). Cells incubated with the R5 medium alone served as negative control, whereas cells stimulated with staphylococcal enterotoxin B (3 µg/mL, Sigma-Aldrich, S4881) served as positive control. Antibodies used for surface and intracellular staining are listed in the **Supplemental Methods** (**SDC**, http://links.lww.com/TP/D252).

### Anti-programmed Cell Death Protein 1 Treatment

Anti-programmed cell death protein 1 (PD-1)antibody (Pembrolizumab, 1 µg/mL, MedChemExpress, HY-P9902) was added on days 4 and 11 simultaneously with IL-2 and IL-7 in conventional and G-Rex expansions. Surface expression of PD-1 was measured on days 0, 10, and 14 by flow cytometry.

### Killing Assay Using Pulsed Phytohemagglutinin Blasts

G-Rex 10 d (D10) expanded cells were labeled with carboxy-fluoresceine-diacetate-succinimidyl-ester (2.5 µM, Invitrogen, Carlsbad, CA, 0850-84) and used as effector cells. To generate phytohemagglutinin (PHA) blasts, autologous PBMCs (500 000 cells/mL) were stimulated with PHA-L (4 µg/mL, Roche Diagnostics, Rotkreuz, Switzerland, 11249738001). On day 3, PHA blasts were harvested, counted and pulsed overnight with 9m127 (2 µg/mL, Eurogentec). Pulsed PHA blasts were then stained with CellTrace Violet (2 µM, Invitrogen, C34557) and used as target cells for killing. Effector cells and target cells were incubated for 6 h at different target:effector ratios (T:E). Autologous PBMCs stained with CellTrace Far Red (1 uM, Invitrogen, C34564) were used for the normalization and acquisition. Specific killing was calculated as previously.^26^

### xCelligence Assay Using BKPyV-infected RPTECs

RPTECs were seeded in E-plate 96 PET (10 000 cells/well; OLS, Bremen, Germany, 300600910) in epithelial cell medium (EpiCM; ScienCell, Carlsbad, 4101) supplemented with 2% fetal bovine serum (FBS, Sigma-Aldrich, F7524). The next day, RPTECs were washed with EpiCM 0% FBS and infected with BKPyV Dunlop (multiplicity of infection 1) for 2 h. Remaining virus in the supernatant were washed away using EpiCM 0.5% FBS. Forty-eight hours postinfection (hpi), G-Rex D10-expanded cells were added to the plate in the R5 medium at different T:E ratios (1:5, 1:10, 1:20, and 1:40). Electrical impedance in cell culture monolayers was continuously measured using xCelligence system until 96 h. Cell index was normalized at 2 hpi.

### Immunofluorescence Staining

RPTECs were harvested at 24 and 48 hpi, seeded on sterile glass coverslips and fixed with 4% paraformaldehyde (Merck, Darmstadt, Germany, 1.04005) in PBS (Sigma-Aldrich, D8537). Cells were permeabilized with 0.2% Triton X-100 (Sigma-Aldrich, 93426) in PBS at room temperaturs for 10 min. Anti-LTag antibody (mIgG2a, Merck, DP02) was used at 1:50 dilution in 3% bovine serum albumin (NEB, B9001S) for 45–60 min at 37 °C. After washing, secondary antibody (goat anti-mIgG2a-Alexa568, Invitrogen, A21134) at 1:600 dilution in 3% bovine serum albumin in PBS and Hoechst 33342 (Sigma-Aldrich, B2261) at 1:50 000 dilution were added for 45–60 min at 37 °C. Coverslips were mounted with ProLong Gold (Invitrogen, P36935). Microscopy images were taken with CELENAX high-content imaging system at 10× magnification.

### IFN-γ Treatment and PD-L1 Staining

RPTECs were infected with BKPyV Dunlop multiplicity of infection 1 in 6 well plate (400 000 cells in 2 mL of EpiCM 0% FBS). At 6 and 36 hpi, IFN-𝛾 (Peprotech, 300-02) was added (final concentrations 1, 10, or 100 U/mL). RPTECs were harvested 48 hpi, stained with anti-PD-L1-antigen-presenting cell (Becton Dickinson, 563741) and fixed with paraformaldehyde 2% in PBS before flow cytometry.

### Statistical Analysis

Data were analyzed with GraphPad Prism software version 10.1.0. Unpaired and 1-sample t-tests were used. Two-sided *P* values of <0.05 were considered statistically significant.

## Results

### Comparing Conventional and G-Rex Expansions of BKPyV-specific T Cells

**FIGURE 1.**
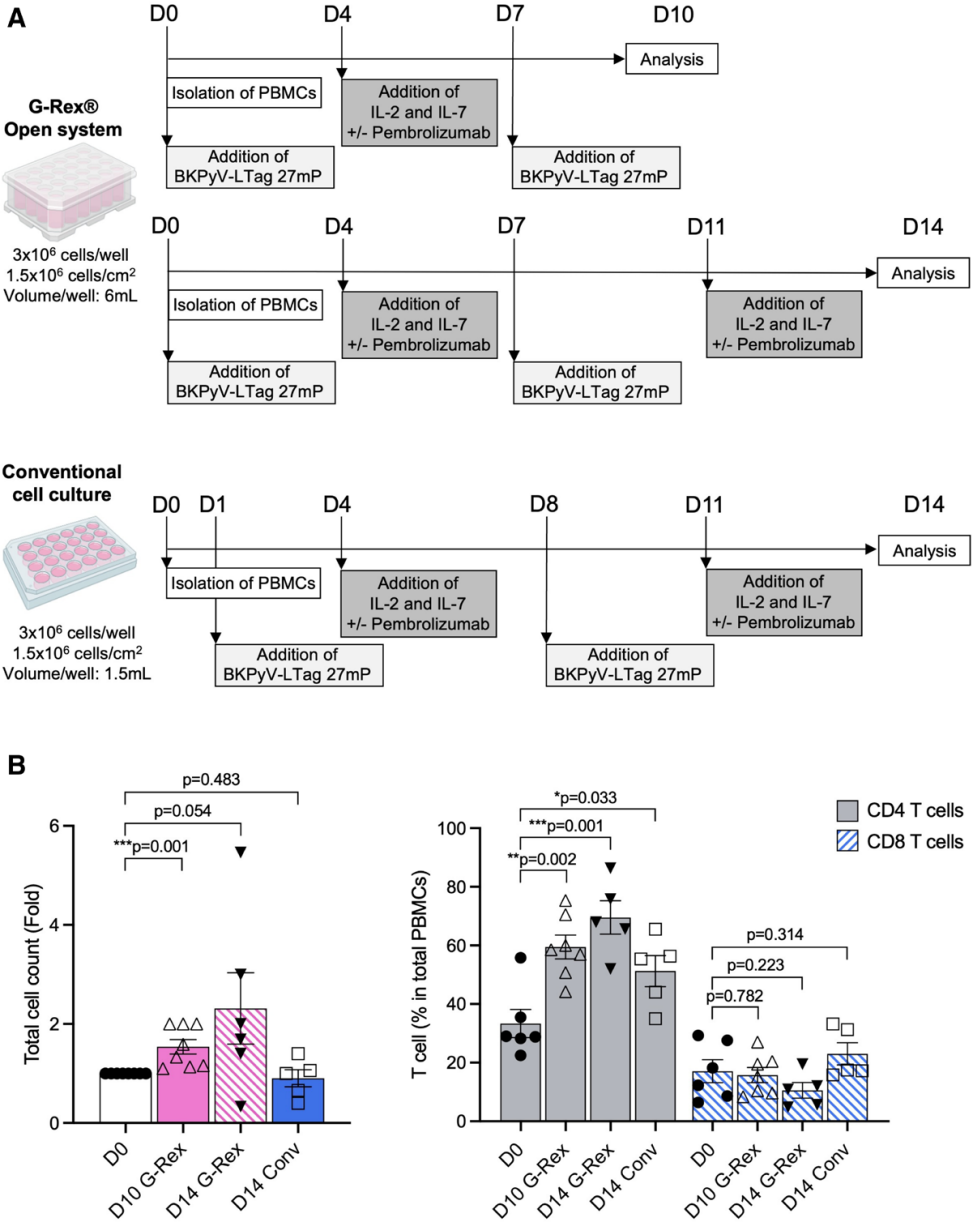
Ex vivo expansion of BKPyV-specific T cells. A, Schematic representation of ex vivo expansions of BKPyV-specific T cells using 24-well cell culture plate and G-Rex open system for 10 and/or 14 d (created with BioRender). B, Total cell count after 10 or 14 d of expansion with G-Rex or conventional cell culture plate. Cell proliferation is normalized to day 0 (left panel). CD4 and CD8 T-cell frequencies in total PBMCs after expansion (right panel). Each symbol represents an individual healthy donor, bar show the mean + SEM. Unpaired 2-tailed t-test. BKPyV, BK polyomavirus; D, day; IL, interleukin; PBMCs, peripheral blood mononuclear cells.

To develop optimized protocols of higher efficacy and yield of BKPyV-specific T cells for functional characterization and adoptive T-cell therapy,^26^ we compared our conventional protocol (conventional cell culture) with the G-Rex open system (G-Rex). PBMCs of healthy blood donors were stimulated with BKPyV LTag-27mP followed by addition of IL-2 and IL-7 and LTag-27mP restimulation (Figure 1A). The overall cell count was higher in G-Rex compared with conventional culture (Figure 1B, left panel) being most significant on day (D)10 (*P* = 0.001). The rate of CD4 T cells was also increased in D10 G-Rex (Figure 1B, right panel) reaching more than 3:1 over CD8 T cells as reported.^26^ We concluded that D10 and D14 G-Rex culture looked promising because of overall higher T-cell yields after ex vivo stimulation than conventional culture.

### Cytokines Production of Expanded BKPyV-specific T Cells

**FIGURE 2.**
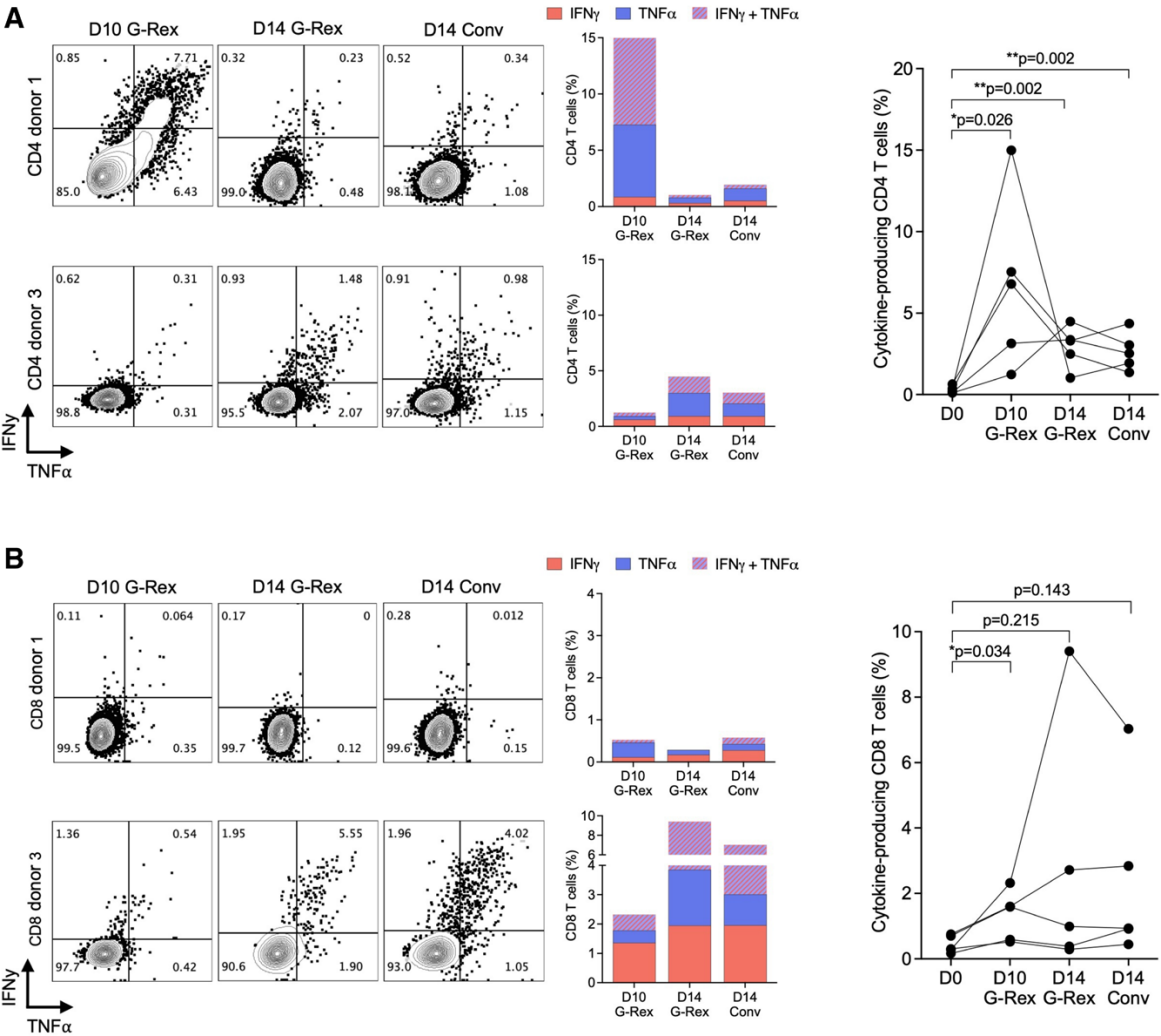
Frequencies of IFN-𝛾 and TNF-α-producing T cells. A, Frequencies of IFN-𝛾 and TNF-α-producing CD4 T cells after 15mP rechallenge when comparing G-Rex system and conventional culture plate. Representative dot plots of 2 healthy blood donors measured by flow cytometry (left panel). Frequency of CD4 T cells producing IFN-𝛾 or TNF-α or both simultaneously (middle panel). Summary results of the 5 donors (right panel). B, Frequencies of IFN-𝛾 and TNF-α-producing CD8 T cells after 9mP rechallenge when comparing G-Rex system and conventional culture plate. Representative dot plots of 2 healthy blood donors (left panel). Frequency of CD8 T cells producing IFN-𝛾 or TNF-α or both simultaneously (middle panel). Combined results of the 5 healthy donors. Unpaired 2-tailed t-test (right panel). 9mP, 9mer pool; 15mP, 15mer pool; IFN-𝛾, Interferon-γ; TNF-α, tumor necrosis factor-α.

The expanded T cells mostly had effector and central memory phenotypes as reported previously.^26^ To investigate the BKPyV-specific T-cell responses, we rechallenged the expansion cultures with BKPyV LTag-15mP and immunodominant LTag-9mP and measured the cytokine production by flow cytometry (Figure 2). The frequency of cytokine-producing CD4 T cells was significantly increased in all expansions compared to day 0. As illustrated for 2 donors, the rate of cytokine-producing CD4 T cells was variable, but overall higher after D10 G-Rex compared with D14 G-Rex or D14 Conv-culture (Figure 2A). Similarly, the frequency of cytokine-producing CD8 T cells showed variability but was higher in D10 G-Rex compared with D0 (Figure 2B). Thus, BKPyV-specific CD4 and CD8 T-cell responses varied across blood donors, with some individuals displaying a robust CD4 but a weak CD8 response, whereas others showed the opposite pattern. We concluded that BKPyV-specific T-cell expansion showed some donor-dependent heterogeneity whereby D10 G-Rex deserved further study because of higher yields and shorter culture.

### Functionality of Expanded BKPyV-specific T Cells

**FIGURE 3.**
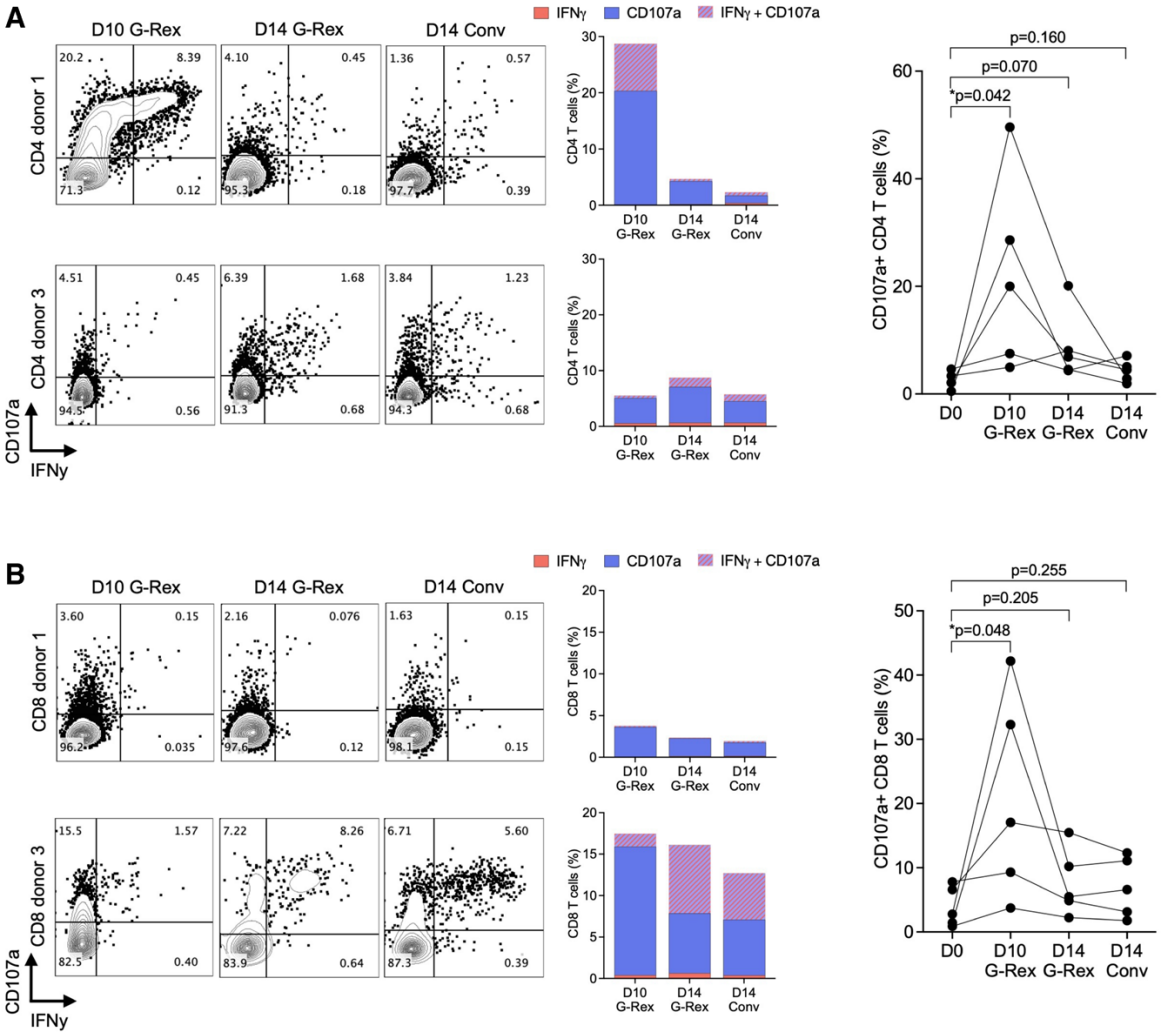
Frequencies of degranulating T cells. A, Representative dot plots of BKPyV-specific CD4 T-cell responses after 15mP rechallenge measured by flow cytometry. Function of expanded T cells was measured as CD107a and IFN-𝛾 expression (left panel). Frequency of degranulating (CD107a^+^) and/or IFN-𝛾-producing CD4 T cells (middle panel). Summary results of 5 healthy donors (right panel). B, Representative dot plots of BKPyV-specific CD8 T-cell responses after 9mP rechallenge measured by flow cytometry. Function of expanded T cells was measured as CD107a and IFN-𝛾 expression (left panel). Frequency of CD8 T cells degranulating (CD107a^+^ cells) and producing IFN-𝛾 alone or in combination (middle panel). Summary results of 5 healthy donors (right panel). Unpaired 2-tailed t-test. 9mP, 9mer pool; 15mP, 15mer pool; BKPyV, BK polyomavirus; IFN-𝛾, interferon-𝛾.

To further characterize the expanded BKPyV-specific T cells, we re-pulsed and measured expression of IFN-𝛾 and CD107a as markers of polyfunctionality (Figure 3). The frequency of degranulating cells (CD107a^+^ cells) was significantly higher in CD4 T cells and in CD8 T cells after D10 G-Rex expansion compared with D14 G-Rex and D14 Conv. As illustrated for 2 donors, the responses in CD4 and in CD8 T cells tended to be variable, but although the rate of IFN-𝛾 -expressing T cells tended to be low, they were mostly co-expressing CD107a (Figure 3, middle panels).

**FIGURE 4.**
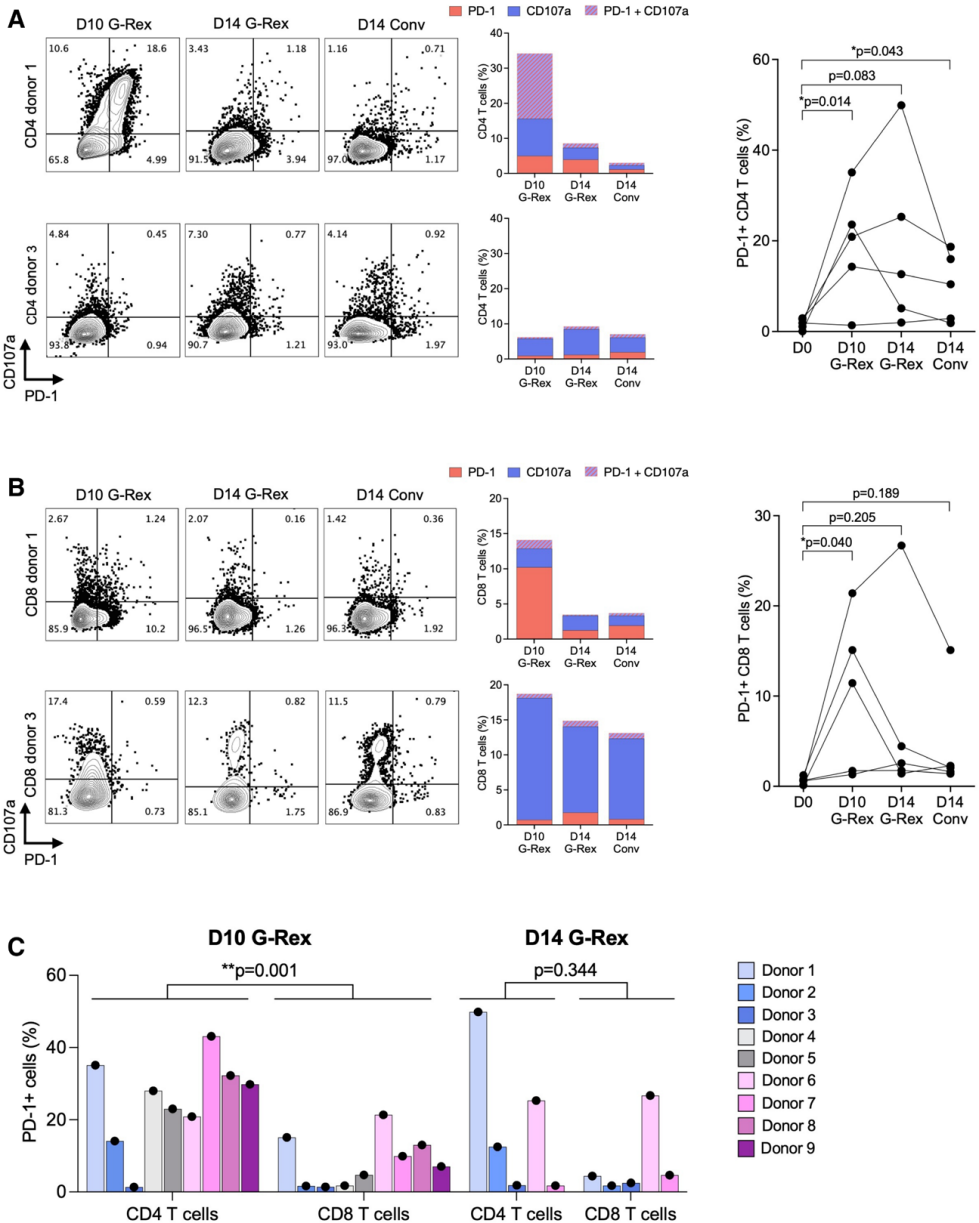
Frequencies of exhausted T cells. A, Representative dot plots of BKPyV-specific CD4 T-cell responses after 15mP rechallenge measured by flow cytometry. Function of expanded T cells was measured as CD107a and PD-1 expression (left panel). Frequency of exhausted (PD-1^+^) and/or degranulating (CD107a^+^) CD4 T cells (middle panel). Summary results of 5 healthy donors (right panel). B, Representative dot plots of BKPyV-specific CD8 T-cell responses after 9mP rechallenge measured by flow cytometry. Function of expanded T cells was measured as CD107a and PD-1 expression (left panel). Frequency of exhausted (PD-1^+^) and degranulating (CD107a^+^) CD8 T cells (middle panel). Summary results of 5 healthy donors (right panel). C, Frequency of T cells expressing PD-1 after 10 or 14 d of expansion with G-Rex. Each donor is shown separately. Unpaired 2-tailed t-test. 9mP, 9mer pool; BKPyV, BK polyomavirus; PD-1, programmed cell death protein 1.

To better understand reasons of heterogeneity, we examined the expression of the immune checkpoint protein PD-1 (Figure 4). As illustrated for 1 donor, a significant population of CD4 T cells having the degranulation marker CD107a also expressed the PD-1 marking cells with exhaustion (Figure 4A), whereas another donor showed only low frequencies of PD-1-expressing CD4 and CD8 T cells (Figure 4A and B). Indeed, a comparison of 9 different blood donors revealed that PD-1 expression appeared to be partly donor-dependent and varied according to the different expansion protocols (Figure 4C). Overall, however, the frequency of PD-1-expressing CD4 and CD8 T cells was significantly increased in D10 G-Rex (Figure 4A and B). The frequency of PD-1-positive T cells was significantly higher in CD4 population compared with CD8 population after D10 G-Rex expansion, but this difference appeared to fade together with the overall responses until day 14 (Figure 4C).

### Cytotoxic Activity of Expanded BKPyV-specific T Cells

**FIGURE 5.**
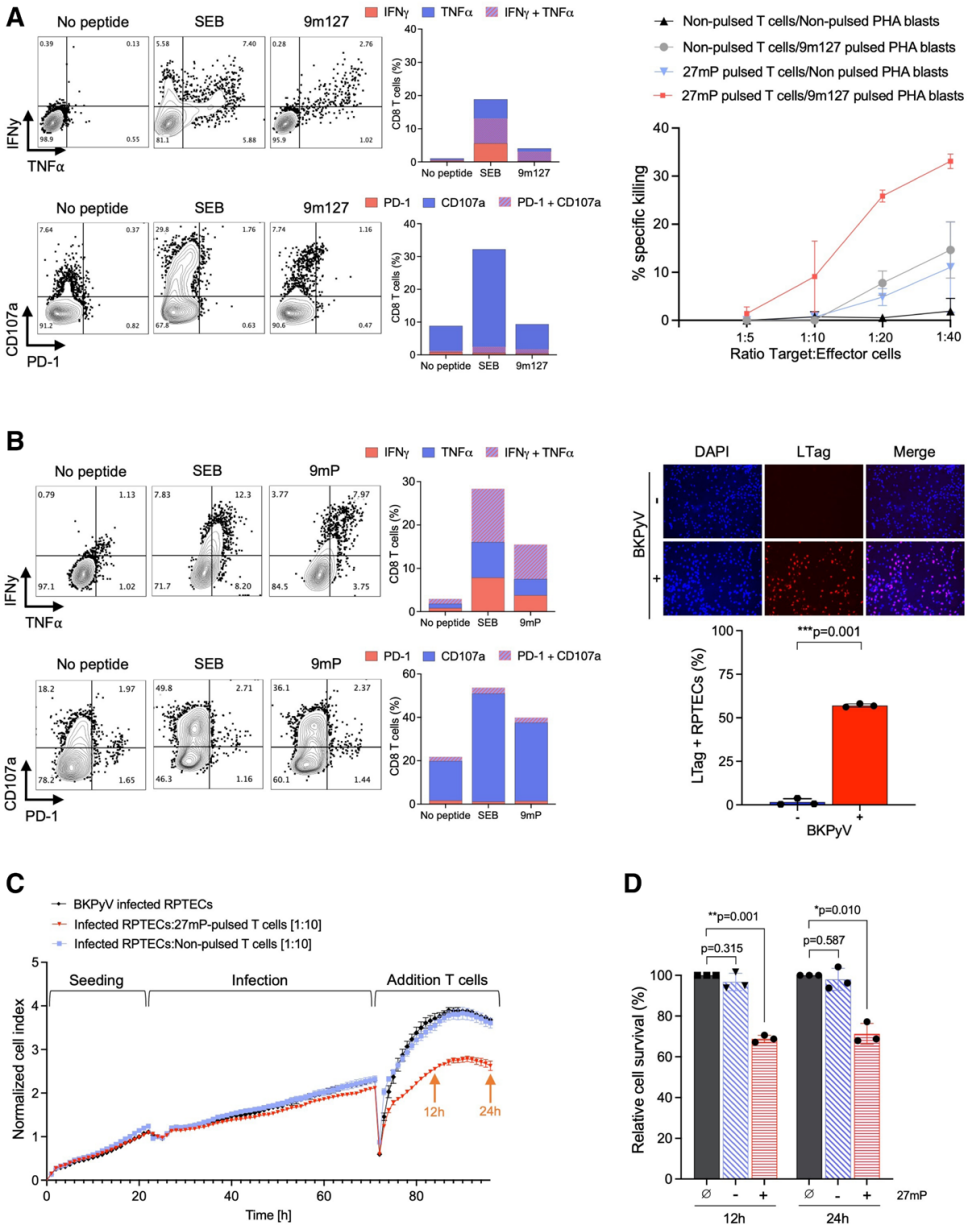
Killing activity of G-Rex 10D expanded BKPyV-specific T cells. A, Characterization of 27mP-pulsed T cells used for killing of 9m127-pulsed PHA blasts. Cells were either not rechallenged (negative control) or rechallenged with SEB (positive control) or 9m127 peptide. Representative dot plots measured by flow cytometry (left panel). Frequency of IFN-𝛾 and/or TNF-α-producing CD8 T cells as well as degranulating and/or PD-1 expressing CD8 T cells (middle panel). 9m127-specific cytotoxic activity of 27mP-pulsed T cells. Nonpulsed T cells were used to determine unspecific killing. Autologous PHA blasts stained with CellTrace violet and pulsed with 9m127 were used as target cells and incubated for 6 h with nonpulsed or 27mP-pulsed T cells (effectors) stained with CFSE. Percentage of target cell killing at different target:effector (T:E) ratios is shown (right panel). B, Characterization of 27mP-expanded T cells used for killing of BKPyV-infected RPTECs. Representative dot plots measured by flow cytometry (left panel). Frequency of IFN𝛾- and/or TNFα-producing CD8 T cells as well as degranulating and/or PD-1 expressing CD8 T cells (middle panel). Expression of LTag on RPTECs 48 hpi with BKPyV Dunlop (MOI 1) measured by immunofluorescence (right panel). Microscopy images taken with CELENAX high-content imaging system at 10× magnitude (top panel). Infection efficiency of RPTECs 48 hpi (bottom panel). C, Cell index of RPTECs before/after infection and after addition of 27mP-pulsed or nonpulsed T cells at 1:10 target:effector ratio using xCELLigence system. D, Survival of BKPyV-infected RPTECs 12 and 24 h post-coculture with nonpulsed or 27mP-pulsed T cells, expressed relative to the condition without T-cell addition (∅). One-sample t-test. Bar show the mean ± SD. 27mP, 27mer pool; 9m127, immunodominant 9mer 127; BKPyV, BK polyomavirus; CFSE, carboxy-fluoresceine-diacetate-succinimidyl-ester; hpi, hours postinfection; IFN-𝛾, interferon-𝛾; LTag, large T-antigen; MOI, multiplicity of infection; PHA, phytohemagglutinin; RPTECs, renal proximal tubule epithelial cells; SEB, staphylococcal enterotoxin B; TNF-α, tumor necrosis factor-α.

To assess the cytotoxic capacity of expanded BKPyV-specific T cells, autologous PHA blasts were pulsed with the immunodominant 9mer-127 (9m127) and cocultured with D10 G-Rex-culture cells (Figure 5). The respective T cells produced IFN-𝛾, tumor necrosis factor α (TNF-α) and degranulated upon 9m127 rechallenge, indicating that BKPyV-specific CD8 T cells were polyfunctional (Figure 5A, left panel). Coculture of the expanded BKPyV-specific T cells with 9m127-pulsed PHA blasts for 6h revealed specific target cell killing, whereas killing was not observed for non-pulsed PHA blasts and only minimal for 9m127-pulsed PHA blasts and nonstimulated T cells, or stimulated T cells and nonpulsed PHA blasts (Figure 5A, right panel). The data indicated the BKPyV-specific 9mer-dependent cytotoxicity of expanded T-cell cultures.

To examine the cytotoxic capacity of expanded BKPyV-specific T cells in a cell culture model of BKPyV nephropathy,^31^ allogeneic primary human RPTECs were infected with BKPyV and used as target cells. At 48 hpi, approximatively 50% of RPTECs were infected and expressed the viral LTag (Figure 5B). BKPyV-specific T cells were expanded for 10 d in G-Rex. The expanded CD8 T cells produced IFN-𝛾 and TNF-α and expressed CD107a upon 9mP rechallenge (Figure 5B). The real-time impedance measured continuously by the xCelligence system revealed that the 27mP-expanded T cells were able to kill BKPyV-infected RPTECs at a T:E ratio of 1:10 in a time-dependent manner (Figure 5C). In contrast, the cell index remained similar for BKPyV-infected RPTECs not adding Tcells or when adding nonpulsed T cells. Thus, the survival of BKPyV-infected RPTECs was reduced by approximately 35% in the presence of 27mP-expanded T cells compared with nonpulsed T cells (Figure 5D). Together, the results indicated functional BKPyV-specific cytotoxicity of ex vivo 27mP-expanded T cells for autologous PHA blasts and for allogeneic BKPyV-infected RPTECs.

### PD-1 Blockade During Ex Vivo Expansion

**FIGURE 6.**
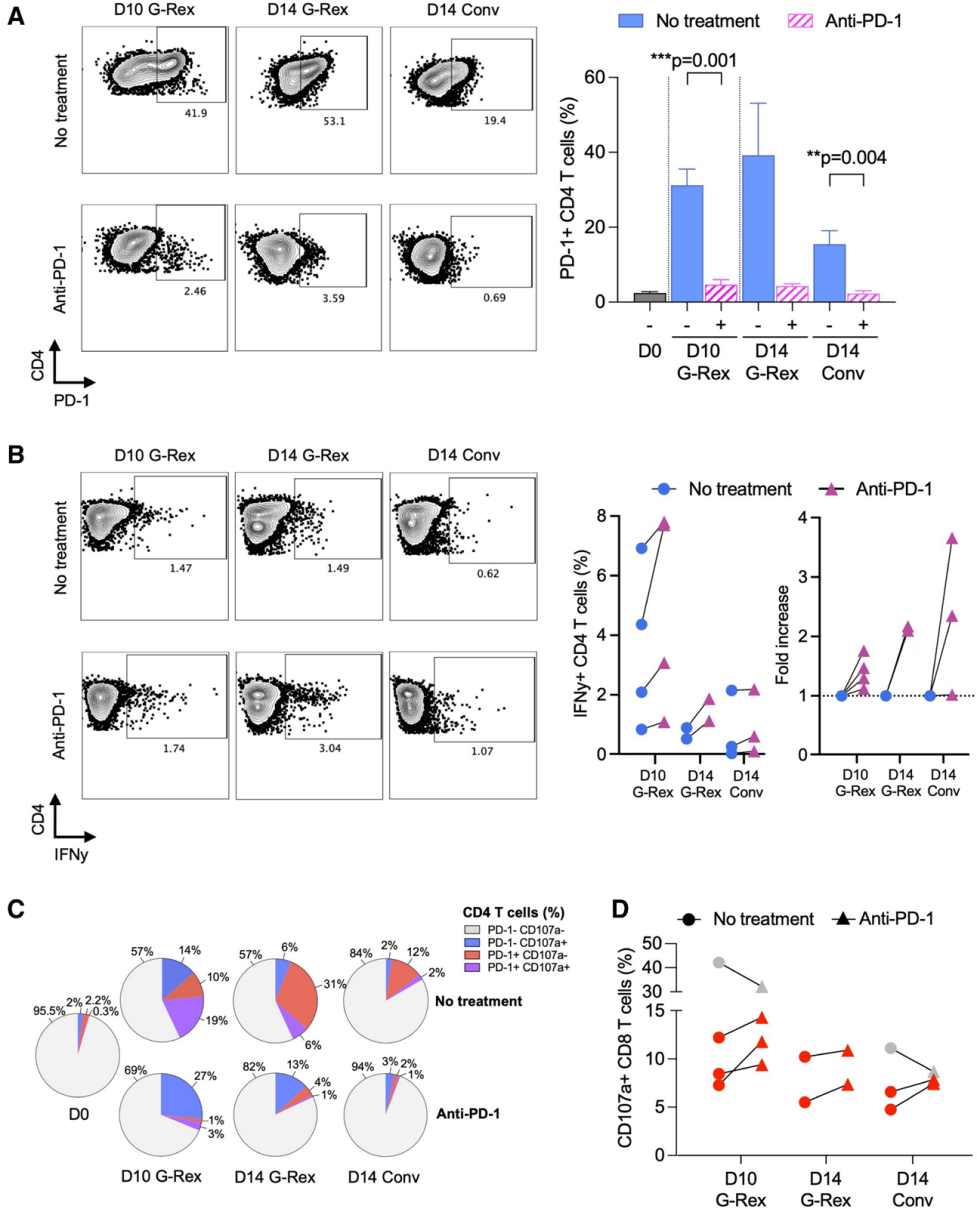
BKPyV-specific T-cell responses following PD-1 blockade. A, PD-1 expression of CD4 T cells after expansion using G-Rex system and conventional culture plate with or without anti-PD-1 treatment (pembrolizumab, 1 µg/mL). Representative dot plots measured by flow cytometry (left panel). Summary results of 3 or 4 donors (right panel). B, Frequency of IFN-𝛾-producing CD4 T cells after 15mP rechallenge using G-Rex system and conventional culture plate with or without anti-PD-1. The background (no rechallenge) has been subtracted to with rechallenge condition to obtain IFN-𝛾-specific response upon 15mP rechallenge. Results are also shown as fold increase (right panel). C, Pie chart summarizing the frequencies of CD107a^+^ and/or PD-1^+^ CD4 T cells after expansion with or without anti-PD-1 treatment and 15mP rechallenge. The mean frequencies of 3 or 4 healthy donors are shown. D, Frequencies of degranulating CD107a^+^ CD8 T cells after expansion with or without anti-PD-1 treatment. Data are highlighted in red when anti-PD-1 treatment induces an increase of the frequency of CD107a^+^ CD8 T cells compared with no treatment condition. One-sample t-test. Bar show the mean + SEM. 15mP, 15mer pool; BKPyV, BK polyomavirus; hpi, hours postinfection; IFN-𝛾, interferon-𝛾; PD-1, programmed cell death protein 1; PD-L1, programmed cell death ligand 1.

To examine the functional impact of PD-1 expression on BKPyV-specific T-cell expansion, PBMCs were pulsed with 27mP and expanded in the presence or absence of a clinically used PD-1-blocking monoclonal antibody, pembrolizumab (Figure 6A). Because CD4 T cells showed a higher level of the exhaustion marker PD-1 compared with CD8 T cells, we focused our analysis on this population. In the absence of pembrolizumab, PD-1 expression ranged from 20% to 40% of CD4 T cells but remained low in the presence of pembrolizumab (Figure 6A). Again, the donor responses were heterogeneous, but the BKPyV-specific CD4 T-cell responses were higher when exposed to pembrolizumab during expansion (Figure 6B). Notably, the fraction of CD4 T cells expressing CD107a without simultaneous PD-1 expression was also increased upon pembrolizumab treatment, in particular in the D10 G-Rex culture (Figure 6C), and some donors also showed an increase of degranulating CD8 T cells upon pembrolizumab treatment (Figure 6D). However, pembrolizumab did not completely abolish PD-1 expression in all donors (**Figure S1, SDC**, http://links.lww.com/TP/D252). We concluded that BKPyV-specific T-cell responses could be enhanced by blocking PD-1 upregulation during ex vivo expansion.

### PD-L1 Expression and Survival of BKPyV-infected RPTECs After IFN-γ Treatment

**FIGURE 7.**
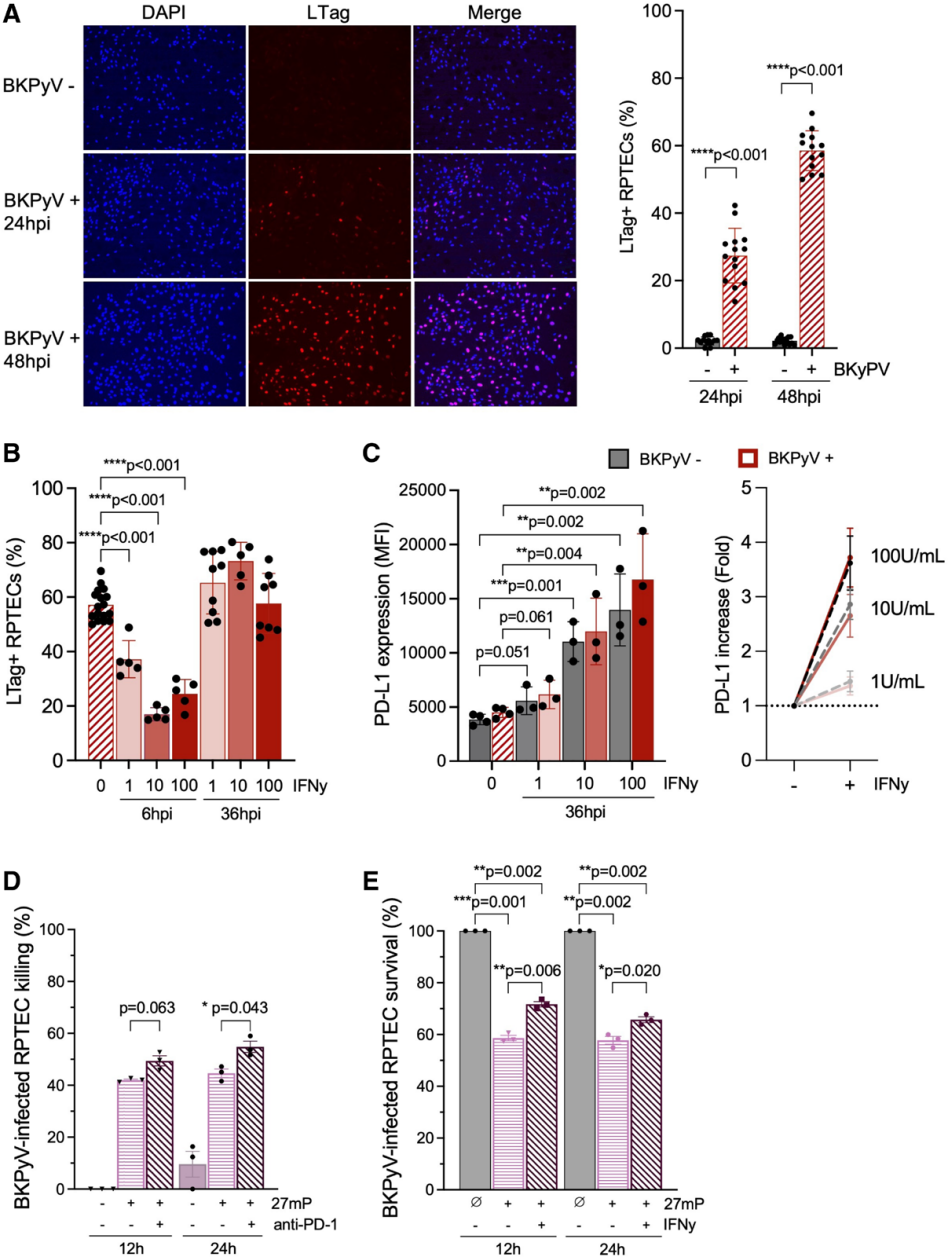
IFN-γ treatment, PD-L1 expression, and viability of BKPyV-infected RPTECs after addition of BKPyV-specific T cells. A, Infection rate of RPTECs with BKPyV Dunlop. Microscopy images of immunofluorescence at 10× magnitude (left panel) and infection rate of LTag-positive cells at 24 and 48 hpi (right panel). B, BKPyV infection rate in response to IFN-γ treatment. Cells were treated with IFN-γ at 6 or 36 hpi with the indicated concentrations and LTag-positive cells were enumerated at 48 hpi. C, PD-L1 expression on RPTECs after IFN-γ treatment. MFI of PD-L1 expression on uninfected and BKPyV-infected RPTECs 48 hpi, with or without IFN-γ treatment (0, 1, 10, or 100 U/mL) at 36 hpi (left panel). Fold change in PD-L1 expression on RPTECs without and with IFN-γ treatment (right panel). D, Effect of anti-PD-1 on killing of BKPyV-infected RPTECs after adding 27mP-pulsed T cells at 48 hpi. Killing rate at 12 or 24 h without (−) or with (+) addition of 27mP-pulsed T cells expanded in the absence (−) or presence (+) of anti-PD-1 pembrolizumab. E, Effect of IFN-γ on viability of BKPyV-infected RPTECs after adding 27mP-pulsed T cells at 48 hpi. BKPyV-infected RPTECs were treated at 36 hpi with solvent (−) or 10 U/mL IFN-γ (+) and the survival rate was measured after 12 or 24 h in the absence (∅) or presence of 27mP-pulsed T cells. Unpaired 2-tailed t-test and 1-sample t-test were used. Bar show the mean + SEM. 27mP, 27mer pool; BKPyV, BK polyomavirus; hpi, hours postinfection; IFN-𝛾, interferon-𝛾; LTag, large T-antigen; MFI, mean fluorescence intensity; PD-L1, programmed cell death ligand 1; RPTECs, renal proximal tubule epithelial cells.

Given the increase in PD-1 expression during ex vivo T-cell expansion, we examined the expression of PD-L1 on RPTECs. The RPTECs were infected with BKPyV and expression of the early viral gene LTag was measured 24 and 48hpi showing infection rates ranging from 20% to 60% (Figure 7A). To mimic inflammatory conditions of BKPyV nephropathy presenting as interstitial nephritis, increasing concentrations of IFN-γ were added at 6 or 36 hpi. Compared with mock treated cells, early addition of IFN-γ significantly decreased the BKPyV infection rate measured at 48 hpi in a dose-dependent manner, whereas addition of IFN-γ at 36 hpi did not affect the rate of LTag-positive cells (Figure 7B). Expression of PD-L1 was detectable on RPTECs and significantly increased in BKPyV-infected and noninfected RPTECs at 48 hpi even when added as late as 36 hpi (Figure 7C). We next evaluated whether pembrolizumab addition during 27mP expansion affected the killing of BKPyV-infected RPTECs. The results showed that killing was increased by anti-PD-1 treated BKPyV-specific T cells compared with mock T cells or untreated BKPyV-infected RPTECs (Figure 7D). Conversely, PD-L1 upregulation by IFN-γ pretreatment at 36 hpi increased the survival of BKPyV-infected RPTECs after addition of BKPyV-specific T cells (Figure 7E). The data support the notion that IFN-γ and PD-1 expression are relevant modulators of the cytotoxic T-cell effector functions in this cell culture model BKPyV nephropathy.

## DISCUSSION

BKPyV-specific adoptive T-cell transfer may shift the balance in favor of BKPyV-specific immune control and holds promise to prevent unfavorable outcomes of BKPyV diseases in patients not responding to reduced immunosuppression.^27-30^ Although recent studies strongly suggest that third-party multi-VSTs are safe and do not increase the risk of acute alloimmune reactions such as graft-versus-host-disease or rejection,^29,30^ their efficacy for BKPyV in KTRs appears to be lower than expected.^32,33^ This could be in part because of continuously administered immunosuppressive drugs,^29^ which may hamper proliferation and antiviral effector functions of transferred T cells in response to BKPyV epitopes.^34^ Our study aiming at developing optimized protocols for ex vivo BKPyV-specific T-cell expansion from healthy blood doors provides the following insights:

First, ex vivo expanded BKPyV-specific T-cell counts were substantially higher in the G-Rex culture compared with conventional culture and reflected increased CD4 T cells and to some extent also CD8 T cells. Despite all donors being BKPyV-IgG seropositive, however, the BKPyV-specific T-cell responses were heterogeneous and varied in magnitude between individuals.

Second, the expanded BKPyV-specific T cells were polyfunctional expressing cytokines such as IFN-γ and TNF-α as well as the degranulation marker CD107a and were cytotoxic for both, autologous PHA blast pulsed with immunodominant BKPyV-9mers or for BKPyV-replicating allogeneic RPTECs. At the same time, a substantial proportion of T cells also expressed the PD-1 marker, with higher levels in the G-Rex cultures at 10 d compared with the 14 d cultures having lower BKPyV-specific responses.

Third, the addition of the immune checkpoint inhibitor, pembrolizumab, during expansion reduced PD-1 expression and increased the overall BKPyV-specific T-cell responses. This observation supported the notion that PD-1 expression is functional and contributes to donor heterogeneity by reducing both, rate and efficacy of BKPyV-specific T-cell responses.

Increased PD-1 expression of ex vivo expanded BKPyV-specific T cells appears to be particularly relevant for patients with BKPyV nephropathy because increased PD-L1 expression has been reported in BKPyV-replicating renal tubules of renal allografts.^35^ This observation may also be relevant to biopsy-proven BKPyV nephropathy presenting as interstitial nephritis, which has been classified in 3 substages: PyVAN stage B1, B2, and B3 according to the 2019 American Society of Transplantation–Infectious Disease Community of Practice guidelines.^6^ These stages aim to capture increasing inflammation in BKPyV-affected tubules associated with progressive renal allograft failure.^36^

Our data emphasize that primary human RPTECs show a low constitutive expression of PD-L1, which could be substantially increased in response to exogenous IFN-γ in a dose-dependent manner. The increase in PD-L1 expression by IFN-γ occurred in both noninfected and BKPyV-infected RPTECs within 12 h, even when IFN-γ was added as late as 36 hpi, a critical timepoint when BKPyV replication is known to switch from early to late viral gene expression encompassing agnoprotein and the capsid proteins Vp1 and Vp2/3.^10,31,37^ The rapid upregulation of PD-L1 in response to IFN-γ could also underlie the flattening out of the killing curve of BKPyV-infected RPTECs at 12 h after the addition of BKPyV-specific T cells in the real-time impedance measurements. Unlike IFN-γ addition at 6 hpi, the rate of BKPyV-infected RPTECs at 48 hpi was not reduced by adding IFN-γ at 36 hpi.

Together, these findings may contribute to the recent results of Khoury and colleagues reporting that only 42% of 38 KTRs showed a decline of BKPyV-DNAemia of >1 log_10_ copies/mL whereby clearance of BKPyV-DNAemia was only observed in 14%.^29^ Importantly, none of 20 KTRs (53%) with biopsy-proven BKPyV-nephropathy showed clinical improvement following multi-VST infusion, suggesting that recovery of advanced stages of BKPyV-nephropathy presenting as interstitial nephritis cannot be easily achieved.^38^

Upregulation of PD-1 on T cells has been linked to significant antigen stimulation and is thought to represent a potential safeguard against immunopathology under physiological conditions following primary infection or vaccination.^39^ However, in the context of prolonged antigen exposure as encountered in malignancies or persisting viral replication such as HIV,^40^ chronic hepatitis C,^41^ or even prolonged cytomegalovirus (CMV) replication,^42^ upregulated PD-1 expression has been linked to loss of multifunctionality and effector functions.^43^ Reversal of this PD-1-mediated exhaustion state in the immunotherapy of malignancies has been the rationale for the successful clinical use of so-called immune checkpoint inhibitors such as pembrolizumab, nivolumab or anti-PD-L1/L2, and avelumab. This may also apply to other viral pathologies including those caused by CMV, Epstein-Barr Virus, adenovirus or other polyomaviruses. Indeed, Cortese et al^44^ reported that pembrolizumab also holds promise in treating progressive multifocal leukoencephalopathy, a devastating brain disease caused by the closely related JCPyV in immunocompromised patients. Moreover, Cortese et al have administered ex vivo expanded BKPyV-specific T cells to treat JCPyV-associated progressive multifocal leukoencephalopathy,^45^ the efficacy of which might be enhanced by pembrolizumab addition as suggested here. Our own study on CMV-specific T cells^46^ revealed that blocking PD-1 signaling by anti-PD-L1/L2 can reverse functional exhaustion in vitro.^47^ However, reversal of exhaustion may be more effective if applied early because longer-term antigen exposure prevents recovery and functional T-cell memory.^48^

Our prior studies indicated that exogenous IL-15 was largely dispensable for the expansion of T cells following 27mP pulse of PBMCs and addition of IL-2 and IL-7, perhaps reflecting local antigen-presenting cell expression of IL-15 and stimulation of the heterotrimeric receptor complexes of IL-15Rα, IL-7R, IL-2Rβ and the common γ-chain CD132. Indeed, Beltra et al^49^ reported in a preclinical murine model that PD-1 expression in response to the lymphocytic choriomeningitis virus gp33 peptide was similarly upregulated by addition of IL-2 or IL-15. Most of the expanded T cells had effector memory and central memory phenotypes.^26^ These findings are consistent with our earlier characterization of BKPyV-specific immunodominant 9mers^23,50^ and the recent report by Vasileiou et al^51^ who actually included rhIL-15 during expansion. However, systematic studies of various cytokine combinations and doses may further improve the functional yield of virus-specific T-cell expansions in cases where ex vivo pembrolizumab is to be avoided. Other experimental approaches suggest that PD-1 expression could be selectively modulated epigenetically, for example, by introducing selective enhancer deletions using Crisp/Cas technology, which in the future may allow for virus-specific T-cell immune control without excess immunopathology.^52^

Importantly, PD-L1 expression on renal tubular epithelial cells has been linked to protection of kidney function from inflammatory injury.^53^ Indeed, the systemic use of immune checkpoint inhibitors for cancer immunotherapy has been associated with acute kidney injury in approximately 5% of patients, many of whom also received additional medications such as proton pump inhibitors, nonsteroidal anti-inflammatory drugs and/or antibiotics.^54^ Thus, the use of PD-1/PD-L1/L2 blockade in transplant patients is strongly discouraged because of the risk of precipitating or exacerbating alloimmune sensitization and graft failure. PD-L1 and L2 expression on tubular epithelial cells not only avert autologous inflammatory injury but are thought to contribute to peripheral tolerance in the alloimmune kidney transplant setting.^55,56^ Perhaps the most striking dilemma arises when solid organ transplantation is complicated by malignancies of poor prognosis, for which immune checkpoint inhibitors have shown significant benefit in nontransplant patients. A recent systematic review of the literature summarized 83 cases of checkpoint inhibitor use for cancers in solid organ transplantation including 43% KTRs.^57^ More than half of the cases suffered from malignant melanoma (N = 46), followed by hepatocellular carcinoma (N = 12) and skin squamous cell carcinoma (N = 10), Merkel cell carcinoma (N = 2), renal cell (N = 1), and urothelial cancer (N = 1). Most patients received anti-PD-L1/L2, pembrolizumab or nivolumab alone, or in combination. Allograft rejection occurred in 40% of patients leading to transplant failure in 71%. Median overall survival was 36 wk and similar in KTRs, whereby death was mostly because of cancer progression in 79% leaving only 19% of patients alive, free from rejection and tumor progression.

Are there ways to overcome these limitations and enhance the efficacy of BKPyV-specific third-party adoptive T-cell therapy for BKPyV replication in KTRs? Perhaps the ambition to target the pathologies of adenovirus, CMV, human herpes virus 6, BKPyV, and by homology JCPyV with 1 magic bullet of multi-VSTs, while logistically and economically attractive, needs to be tempered and instead refocus on specific patient and transplant constraints to convincingly increase efficacy. For BKPyV in KTRs, the recent studies of Khoury et al.^29^ and Chandraker et al^30^ suggest that donor heterogeneity and timing remain important obstacles for achieving higher efficacy. One interesting variable is to ensure the BKPyV-specific serostatus of the donors and perhaps ensure dual serotype-specific IgG to BKPyV serotype-1 and serotype-4 which would cover 90% of detected BKPyV-subtypes.^58,59^ Although the BKPyV-serotypes reflect differences in neutralizing antibodies,^1,59^ our recent study also suggests differences in immunodominant 9mers relevant for BKPyV-specific T-cell responses.^4^ Increasing the rate and the quality of HLA-matching between the administered T-cell preparation and the renal allograft could be optimized whereby including HLA class 1 presenting previously characterized immunodominant 9mers to CD8 T cells represent a key opportunity.^23,26^ Also, adoptive T-cell therapy protocols need to be developed for safe reduction of immunosuppression permitting antiviral T-cell effector functions without causing alloimmune damage. Finally, as suggested by the present study, PD-1 blockade during expansion or before administration might be an effective way to avoid adverse systemic effects of immune checkpoint inhibitors while by the same token, increasing the cytotoxic efficacy on the target BKPyV-replicating renal tubular cells, even in the inflammatory context of BKPyV nephropathy of PyVAN stage B. Precedent for this kind of preinfusion modification is given by T-cell depletion protocols in allogeneic hematopoietic cell transplantation.

Our study has several limitations. First, we performed ex vivo T-cell expansion only in a limited number of healthy donors and the generalizability of our observations could be questioned. However, the results are consistent with our earlier observations^25,34^ and those by others.^24^ Importantly, our donors had diverse HLA types and were well characterized with respect to their positive BKPyV serostatus. Second, although our study demonstrates the feasibility and efficacy of the G-Rex system for ex vivo BKPyV-specific T-cell expansion, the exact mechanisms driving increased PD-1 expression during this process remain unclear. Further investigations will improve the factors contributing to this upregulation, including time and dosing of antigen exposure, T-cell receptor signaling, cytokines and metabolic changes. Importantly, antigen-specific polyfunctional T-cell responses can be detected following ex vivo expansion even in the presence of upregulated PD-1. This perhaps physiological response to antigen-specific expansion may induce exhaustion not during primary characterization of the T-cell preparation but only after encountering the respective PD-L1 or L2 in the affected nephron. While this is highly likely to increase tolerability and safety, PD-1 upregulation may reduce the efficacy for advanced virus pathologies such as BKPyV-nephropathy. Third, we have not fully elucidated the maximal duration of the antiexhaustion effects of pembrolizumab. The results of the 10-d expansion protocol indicated that adding pembrolizumab on day 4 lasted for at least 6 d. This suggests that pembrolizumab effects ex vivo are relatively durable and thereby could provide a rationale for clinical trials within this time frame.

In conclusion, donor variability and PD-1 upregulation may contribute to reduced efficacy of adoptive third-party T-cell therapy, which is potentially amenable to several optimization steps for future therapeutic strategies.

## Supplementary Material


