# Supplementary material for: Donor Variability and PD-1 Expression Limit BK Polyomavirus-specific T-cell Function and Therapy
Source: Transplantation. 2025 Apr 9;109(9):1526–39. doi: 10.1097/TP.0000000000005399 (PMC12366737; doi:10.1097/TP.0000000000005399)
Supplement: Supplementary file 1 [file tpa-109-1526-s001.pdf]

SUPPLEMENTAL DIGITAL CONTENT

Donor Variability and PD-1 Expression Limit BK Polyomavirus-Specific T-Cell Function and Therapy

Maud Wilhelm[1], Amandeep Kaur[1], Anne Geng[1], Marion Wernli[1], Hans H. Hirsch[1]
[1]Transplantation & Clinical Virology, Department Biomedicine, University of Basel, Basel, Switzerland

Correspondence:
Professor Hans H. Hirsch, M.D., M.Sc.
Transplantation & Clinical Virology
Department Biomedicine, University of Basel, Petersplatz 10
CH-4009 Basel, Switzerland
Phone: +41 61 207 3266
Fax: +41 61 207 3283
Email: hans.hirsch@unibas.ch

Supplementary methods:

BKPyV-derived peptides and cell culture

All peptides were >70% pure and dissolved in dimethyl sulfoxide (DMSO, 10mg/mL, Eurogentec, Seraing, Belgium). The 27mer pool (27mP) consisted of 34 peptides overlapping by 8 amino acids (aa) covering the entire BLPyV LTag (Dunlop strain)[1]. The 15mer pool (15mP) consisted of 180 peptides of 15 aa in length overlapping by 11 aa and also covering BKPyV LTag[2]. The 9mer pool (9mP) consisted of 97 immunodominant 9mers within BKPyV LTag[1,3,4]. PBMCs were isolated on Ficoll density gradients (Lymphoprep, Alere Technologies AS, Wädenswill, Switzerland, 1114545). Cells were cultured in R5 RPMI medium (Sigma-Aldrich, St-Louis, Missouri, R2405) supplemented with human serum (5%, Sigma-Aldrich, St-Louis, Missouri, H4522), and Penicillin/streptomycin (1%, Sigma-Aldrich, St-Louis, Missouri, P0781).

Flow cytometry analysis

For extracellular staining, the following antibodies were used: CD4 (BD, 562424), CD8 (BioLegend, San Diego, United States, 301012), PD-1 (BD, 557946), CD107a (BD, 561348) and IgG1 isotype control (BD, 558904). For intracellular staining, the following antibodies were used:

IFNγ (BD, 341117) and TNFα (BD, 554512). Flow cytometry acquisition was performed using a Fortessa cytometer and analyzed by FlowJo (version 10.10.0).

Table S1: Characteristics of healthy donors

	HD1		HD2		HD3		HD4		HD5		HD6		HD7		HD8		HD9	
Fresh or frozen cells	Fresh blood		Fresh blood		Fresh blood		Frozen PBMCs		Fresh blood		Frozen PBMCs		Frozen PBMCs		Frozen PBMCs		Fresh blood	
Experiments	Comparison expansion, PD-1 blockade		Comparison expansion		Comparison expansion, Killing assay infected RPTECs		Killing assay Plused PHA blasts		Killing assay infected RPTECs		Comparison expansion, PD-1 blockade		PD-1 blockade		Comparison expansion, PD-1 blockade		Killing assay infected RPTECs, PD-1 blockade	
HLA-A	02	23	03	68	02	02	01	03	01	02	02	66	01	30	02	11	NA	NA
HLA-B	27	57	07	27	07	15	07	08	08	40	08	62	08	13	18		NA	NA
BKPyV gI IgG, nOD	1.642		0.395		1.115		0.334		0.173		0.271		0.235		0.820		3.611	
BKPyV gII IgG, nOD	1.167		0.112		0.334		0.084		0.041		0.132		0.098		0.121		1.594	
BKPyV gIII IgG, nOD	0.811		0.253		0.030		0.041		0.043		0.150		0.114		0.042		0.317	
BKPyV gIV IgG, nOD	1.082		1.020		0.223		0.069		0.145		0.066		0.137		0.123		1.109	
JCPyV IgG, nOD	0.805		0.445		0.066		0.019		2.097		0.895		0.426		0.149		0.121	

This table provides an overview of the characteristics and experimental details of the 9 healthy blood donors tested in this study. The table includes the following information: frozen of freshly isolated PBMCs, HLA type and BKPyV IgG levels and JCPyV IgG levels measured by VLP-Elisa (*normalized Optical density >0.1 of 1:200 diluted plasma is considered positive)
BKPyV, BK polyomavirus; gt, genotype; HD, healthy donor; HLA, human leukocyte antigen; IgG, immunoglobulin G; JCPyV, JC polyomavirus; NA, not assessed; nOD, normalized optical density.

Figure S1: Suppression of PD-1 on expanded T-cells by pembrolizumab.

Frequency of PD-1+ CD4 T-cells after expansion with or without anti-PD-1 treatment. Results of 4 donors are shown. The refractory response of Donor 7 is highlighted in the indicated culture conditions.

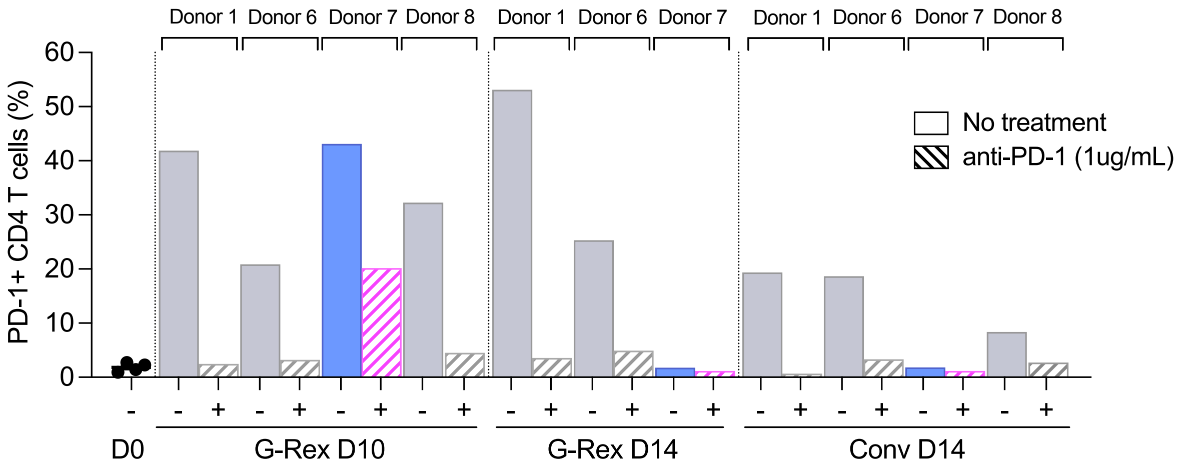

1. Wilhelm M, Kaur A, Wernli M, Hirsch HH. BK Polyomavirus-Specific CD8 T-Cell Expansion In Vitro Using 27mer Peptide Antigens for Developing Adoptive T-Cell Transfer and Vaccination. *J Infect Dis.* 2021;223(8): 1410-1422.
2. Binggeli S, Egli A, Schaub S, et al. Polyomavirus BK-specific cellular immune response to VP1 and large T-antigen in kidney transplant recipients. *Am J Transplant.* 2007;7(5): 1131-1139.
3. Durairaj J, Follonier OM, Leuzinger K, et al. Structural implications of BK polyomavirus sequence variations in the major viral capsid protein Vp1 and large T-antigen: a computational study. *mSphere.* 2024;9(4): e0079923.
4. Leboeuf C, Wilk S, Achermann R, et al. BK Polyomavirus-Specific 9mer CD8 T Cell Responses Correlate With Clearance of BK Viremia in Kidney Transplant Recipients: First Report From the Swiss Transplant Cohort Study. *Am J Transplant.* 2017;17(10): 2591-2600.
